# Social Engagement, Isolation, and Loneliness Among Diverse Caregiving Populations: Insights From National Data

**DOI:** 10.1093/geroni/igaf122.730

**Published:** 2025-12-31

**Authors:** Yiqing Qian, Mara Rosenberg, Christine Ritchie

## Abstract

Social isolation and loneliness are critical issues that shape the well-being of older adults and their family caregivers. Despite growing scientific inquiries into isolation and loneliness since the COVID-19 pandemic, these psychosocial challenges remain underexplored in family caregiving research. This symposium draws on multiple nationally representative datasets to examine how social connection, or lack thereof, influences caregiver experiences and mental health. Together, these papers explore the intersections of social engagement, isolation, and stress among diverse caregiving populations. First, Dr. Rosenberg presents data from the National Study of Caregiving (NSOC) to demonstrate the associations between caregiver participation in social activities and burden. Next, we examine the isolation and mental health of two subgroups of family caregivers for older adults. Dr. Pomeroy focuses on dementia caregivers, a subgroup of caregivers who bear high intensity and burden of care, to characterize how their social isolation and loneliness experiences differ by gender. Linking NSOC with the National Health and Aging Trends Study, Dr. Elmore focuses on understudied long-distance caregivers and examines the association between proximity and mental health. Lastly, Dr. Li presents a comparative analysis between caregivers and non-caregivers in the National Poll on Healthy Aging, demonstrating that despite the high prevalence of loneliness during the COVID-19 pandemic, caregivers are still less likely to use mental health self-care services. Overall, these papers explore caregivers’ distinct social experiences and offer potential targets to improve caregiver well-being. As the discussant, Dr. Kotwal will synthesize these presentations and propose targets for research, policy, and practice.

